# Comparative analysis of urinary antibiograms in community pediatric and geriatric populations in British Columbia, Canada

**DOI:** 10.14440/bladder.2025.0014

**Published:** 2025-10-06

**Authors:** Roxanna S. D. Mohammed, Eugene Y. H. Yeung

**Affiliations:** 1 Department of Obstetrics and Gynaecology, Faculty of Medicine, University of British Columbia, Vancouver, British Columbia V6H 3N1, Canada; 2 Department of Pathology and Laboratory Medicine, Faculty of Medicine, University of British Columbia, Vancouver, British Columbia V5Z 1M9, Canada; 3 Continuing Pharmacy Professional Development, Faculty of Pharmaceutical Sciences, University of British Columbia, Vancouver, British Columbia V6T 1Z3, Canada; 4 School of Medicine, Simon Fraser University, Surrey, British Columbia V3T 0A3, Canada

**Keywords:** Geriatrics, Pediatrics, Urine culture, Antibiogram, Antimicrobial susceptibility testing, Ciprofloxacin

## Abstract

**Background:**

The Clinical and Laboratory Standards Institute (CLSI) recommends the development of enhanced antibiograms specifically for elderly patients (≥65 years) to address the needs of long-term care facilities and to account for the anatomical sites from which specimens are collected, thereby facilitating antimicrobial stewardship. Similarly, the Canadian Pediatric Society advocates developing local, age-specific antibiograms to guide antimicrobial selection for targeted infections.

**Objective:**

This study aimed to develop antibiograms based on uropathogens identified at LifeLabs in British Columbia (BC), Canada.

**Methods:**

Urinary specimens from pediatric (<18 years) and geriatric (≥65 years) patients were collected and processed at LifeLabs, a community laboratory network comprising 129 collection centers across BC, between October 1, 2023, and September 30, 2024. Urinary antibiograms for both groups were developed in accordance with CLSI guidelines.

**Results:**

Among the 13,870 pediatric specimens, the most common uropathogen was *Escherichia coli* (13.7%), followed by *Enterococcus faecalis* (2.0%), *Proteus mirabilis* (1.1%), *Streptococcus agalactiae* (0.9%), *Staphylococcus saprophyticus* (0.9%), and *Klebsiella pneumoniae* (0.5%). Among the 148,480 geriatric specimens, the most common uropathogen was *E. coli* (17.1%), followed by *E. faecalis* (3.6%), *K. pneumoniae* (3.4%), *S. agalactiae* (2.0%), *P. mirabilis* (1.3%), and *Pseudomonas aeruginosa* (0.8%). Among the routine antimicrobials tested, ciprofloxacin consistently demonstrated significantly different susceptibility rates (*p*<0.05) between the pediatric and geriatric groups: *E. faecalis* (96.3% vs. 81.4%), *E. coli* (73.7% vs. 67.3%), and *P. mirabilis* (92.6% vs. 84.8%).

**Conclusion:**

The distribution of common uropathogens and their susceptibilities differed between pediatric and geriatric groups, supporting the need for age-specific antibiograms in community settings. Ciprofloxacin demonstrated lower susceptibility to the predominant uropathogens in elderly patients. Community antimicrobial stewardship teams should acknowledge these differences to better prioritize interventions tailored to each age group.

## 1. Introduction

The 2022 Clinical and Laboratory Standards Institute (CLSI) M39 guideline, “Analysis and Presentation of Cumulative Antimicrobial Susceptibility Test Data,” recommended the development of antibiograms for long-term care facilities (LTCFs).^1^ The CLSI acknowledged the challenges in developing an LTCF-specific antibiogram, such as outsourcing testing to multiple microbiology laboratories within the same facility, the limited number of isolates available in each community laboratory, and the lack of clear ownership for antibiogram preparation. Rather than solely extrapolating data from hospital inpatients, who may have higher resistance rates than community patients, the CLSI recommended preparing antibiograms based on patients in the community aged 65 years and older. LifeLabs in British Columbia (BC), Canada, is uniquely positioned to address these challenges, as it is a community laboratory network with 129 collection centers across both rural and urban communities in BC. It maintains an extensive internal surveillance database that can be leveraged to inform public health and antimicrobial stewardship efforts.

The CLSI also recommended the development of enhanced antibiograms to address specific clinical questions and guide empirical antimicrobial therapy in selected patient populations or infection types, provided that sufficient isolates have been tested to ensure reasonable statistical validity.^1^ LifeLabs in BC has the expertise and adequate sample size to create such syndromic antibiograms specific to urinary tract infections (UTIs) in community settings.^2^ The selection of empirical antimicrobials for UTIs in primary care is unclear in the guidelines issued by the Government of BC, as they simply refer readers to treatment resources provided through local hospitals, health authorities, and laboratories.^3^ There are also growing concerns regarding rising antimicrobial resistance among uropathogens in older adults,^4^ possibly due to the overtreatment of asymptomatic bacteriuria in this population.^5^,^6^ Therefore, it is important to develop a community-specific antibiogram for UTIs in older adults to illustrate the current local resistance rate.

The Canadian Pediatric Society advocates for laboratories to produce local, age-specific antibiograms to guide antimicrobial selection for targeted infections (*e.g*., UTIs), as using antimicrobial resistance patterns from other countries or adult patients may overestimate resistance.^7^ Antimicrobial stewardship teams in other countries also call for pediatric-specific antibiograms.^8^ Pediatric urinary antibiogram data can be compared with geriatric urinary antibiogram data to determine differences in microorganism susceptibility rates, thereby justifying the need for specific antibiograms for each age group. This study aimed to create antibiograms based on uropathogens identified at LifeLabs, BC, Canada, harvested from pediatric and geriatric groups within the communities. The generated data will help antimicrobial stewardship teams provide guidance to community healthcare providers who manage one or both patient groups.

## 2. Materials and methods

### 2.1. Data collection and analysis

The data collection method was adapted from our previously published study on an enhanced antibiogram.^2^ A retrospective analysis was performed on all urine cultures processed and analyzed at LifeLabs, a community laboratory network with 129 collection centers across BC, from specimens taken between October 01, 2023, and September 30, 2024, using the Microbiology Electronic Worksheet System (MEWS; version 5.00.267, LifeLabs, Canada). One year of urinary specimen data from patients of all ages and genders were collected (*n* = 373,068), of which 13,870 and 148,480 specimens were from the pediatric (<18 years) and geriatric (≥65 years) groups, respectively. The antibiograms were developed in accordance with the CLSI M39 guideline,^1^ which recommends the inclusion of:

(i) At least 1 year of data
(ii) Diagnostic isolates only, not surveillance isolates
(iii) Only the first isolate of a species per patient, excluding duplicates
(iv) At least 30 isolates per species to meet the minimum sample size
(v) Only antimicrobial agents routinely tested, excluding supplemental agents
(vi) Both suppressed and unsuppressed antimicrobial susceptibility testing (AST) results were included to minimize bias from selective reporting rules
(vii) Only the percentage of susceptible isolates, excluding intermediate and susceptible dose-dependent results.

GraphPad Prism Version 6.0c (GraphPad Software Incorporation, USA) was employed to perform statistical analyses, including Chi-squared tests to assess categorial differences between groups, with *p*<0.05 considered statistically significant.

### 2.2. Identification of microorganisms in urine culture

The method for identifying microorganisms in urine culture was established in our previous study.^2^ Briefly, urine specimens were inoculated using loops onto BD BBL™ CHROMagar™ (BD, USA) and Sheep Blood Agar Base with 5% Sheep Blood (Oxoid, Canada) plates. These plates were incubated under aerobic conditions and read twice, at 18 and 36 h of incubation. Only microorganisms with a minimum of 10 colonies (equivalent to approximately 1 × 10^7^ colony-forming units/L in routine or special cultures) were considered significant for growth and proceeded to the identification stage. A mixture of three or more different types of microorganisms was interpreted as contamination and, therefore, was not further processed. For identification, microorganisms were isolated and speciated using the VITEK2 System (bioMérieux, USA) and matrix-assisted laser desorption/ionization time-of-flight mass spectrometry (Bruker Daltonics GmbH & Co. KG, Germany), according to the manufacturers’ instructions. The identification results were recorded in the MEWS software.

### 2.3. AST

The AST method was described in detail in our previous study.^2^ Briefly, suspensions of microorganisms were loaded in the VITEK 2 System for AST according to the manufacturer’s instructions, which had been previously validated using CLSI-approved methods.^9^ The CLSI M100 clinical breakpoints for the zone of inhibition and minimum inhibitory concentration were used to determine susceptibility. The AST results were recorded in the MEWS software. However, not every microorganism identified had the full AST panel performed and reported. For instance, *Streptococcus agalactiae* and *Staphylococcus saprophyticus* have predictable susceptibility profiles that do not require testing unless specifically requested by the ordering clinician; therefore, their AST results were not included in the antibiograms to avoid selection bias, in accordance with the CLSI guideline.^1^,^9^ Some microorganisms grew on primary culture plates but failed to grow on the media used for susceptibility testing. Fosfomycin testing was performed only if specifically requested or when no other oral antimicrobial option was available. LifeLabs typically did not perform routine cephalosporin AST when evidence of extended-spectrum β-lactamases (ESBL) production was present in susceptibility patterns, such as resistance to any third-generation cephalosporin.

## 3. Results

### 3.1. Identification of microorganisms in urine culture

Among the 13,870 pediatric urinary specimens, the most common uropathogen was *Escherichia coli* (13.7%), followed by *Enterococcus faecalis* (2.0%), *Proteus mirabilis* (1.1%), *S. agalactiae* (0.9%), *S. saprophyticus* (0.9%), and *Klebsiella pneumoniae* (0.5%). Among the 148,480 geriatric urinary specimens, the most common uropathogen was *E. coli* (17.1%), followed by *E. faecalis* (3.6%), *K. pneumoniae* (3.4%), *S. agalactiae* (2.0%), *P. mirabilis* (1.3%), and *Pseudomonas aeruginosa* (0.8%) (Table 1). *P. aeruginosa* was identified in 29 (0.2%) pediatric specimens, but was not among the most common uropathogens in this group. *S. saprophyticus* was identified in 64 (0.04%) geriatric specimens, but was not among the most common uropathogens in this group.

**Table 1:** Most common uropathogens identified in urine specimens at LifeLabs in British Columbia (October 01, 2023–September 30, 2024)

Age group	Uropathogen	Specimens (*n*)	Percentage
Pediatric (<18 years)	No significant growth	11,131	80.25
	*Escherichia coli*	1906	13.74
	*Enterococcus faecalis*	279	2.01
	*Proteus mirabilis*	158	1.14
	*Streptococcus agalactiae*	129	0.93
	*Staphylococcus saprophyticus*	127	0.92
	*Klebsiella pneumoniae*	63	0.45
	Total	13,870	100.00
Geriatric (≥65 years)	No significant growth	102,521	69.05
	*Escherichia coli*	25,422	17.12
	*Enterococcus faecalis*	5358	3.61
	*Klebsiella pneumoniae*	5097	3.43
	*Streptococcus agalactiae*	2901	1.95
	*Proteus mirabilis*	1991	1.34
	*Pseudomonas aeruginosa*	1189	0.80
	Total	148,480	100.00

### 3.2. AST

Table 2 presents the pediatric and geriatric antibiograms based on uropathogens identified from urinary specimens. The findings revealed that *P. aeruginosa* was not among the most common uropathogens in the pediatric group and therefore was not included in the antibiograms. However, in the pediatric group, *P. aeruginosa* demonstrated a 96.2% susceptibility to ciprofloxacin and a 100% susceptibility to gentamicin, based on 26 and five isolates tested, respectively.

**Table 2:** Antibiogram of uropathogens identified in urine specimens at LifeLabs in British Columbia, across pediatric and geriatric age groups (October 01, 2023–September 30, 2024)

Age group	Uropathogen	AST (*n* [% susceptibility])										

		Ampicillin	Cefazolin^a^	Ceftriaxone	Ciprofloxacin	Fosfomycin	Gentamicin	Nitrofurantoin	Sulfamethoxazole– trimethoprim	Tetracycline	Meropenem	Ceftazidine
Pediatric (<18 years)	*Escherichia coli*	1466 (61%)	1316 (99%)	1466 (90%)	1456 (74%)	1461 (99%)	1466 (92%)	1466 (99%)	1466 (79%)	1459 (79%)	1466 (100%)	NA
	*Enterococcus faecalis*	246 (100%)	R	R	245 (96%)	NA^b^	NA	246 (99%	R	246 (22%)	NA	R
	*Proteus mirabilis*	135 (78%)	53 (100%)	135 (97%)	135 (93%)	NA	135 (96%)	R	135 (84%)	R	135 (100%)	NA
	*Streptococcus agalactiae*	AST was not routinely performed										
	*Staphylococcus saprophyticus*	AST was not routinely performed										
	*Klebsiella pneumoniae*	R	53 (100%)	56 (95%)	55 (95%)	NA	56 (100%)	56 (23%)	56 (96%)	55 (96%)	56 (100%)	NA
Geriatric (≥65 years)	*Escherichia coli*	14, 991 (61%)	13,132 (99%)	14,992 (88%)	14,992 (67%)	101 (92%)^b^	14,991 (93%)	14,992 (97%)	14,992 (82%)	14,992 (79%)	14,983 (100%)	NA
	*Enterococcus faecalis*	3753 (100%)	R	R	3753 (81%)	101 (92%)^b^	NA	3753 (99%)	R	3753 (25%)	NA	R
	*Klebsiella pneumoniae*	R	3059 (100%)	3246 (94%)	3246 (88%)	NA	3246 (98%)	3246 (29%)	3246 (93%)	3246 (25%)	3245 (100%)	NA
	*Streptococcus agalactiae*	AST was not routinely performed										
	*Proteus mirabilis*	1402 (77%)	1351 (100%)	1402 (97%)	1402 (85%)	NA	1402 (92%)	R	1402 (83%)	R	1401 (100%)	NA
	*Pseudomonas aeruginosa*	R	R	R	771 (93%)	R	131 (98%)^b^	R	R	R	770 (96%)	771 (96%)

Notes:

a The uncomplicated cystitis cefazolin breakpoint (minimum inhibitory concentration ≤16 µg/mL) was applied. Cefazolin susceptibility results were extrapolated to oral cephalosporins in accordance with the Clinical and Laboratory Standards Institute M100 guideline. Cefazolin testing was not performed at LifeLabs when resistance patterns suggested the presence of ESBL.

b This antimicrobial is not included in the LifeLabs routine first-line testing panel for this microorganism. “NA” indicates data not available due to limited sample size, whereas “R” denotes intrinsic resistance. n is the number of isolates tested.

Abbreviation: AST Antimicrobial susceptibility testing.

Among the routine antimicrobials tested, ciprofloxacin consistently demonstrated significantly different susceptibility rates (*p*<0.05) between the pediatric and geriatric groups: *E. faecalis* (pediatric 96.3% vs. geriatric 81.4%), *E. coli* (pediatric 73.7% vs. geriatric 67.3%), and *P. mirabilis* (pediatric 92.6% vs. geriatric 84.8%). Compared with the pediatric group, the geriatric group also exhibited a trend toward a reduced ciprofloxacin susceptibility in *K. pneumoniae* (pediatric 94.5% vs. geriatric 81.3%) and *P. aeruginosa* (pediatric 96.2% vs. geriatric 92.7%), although these differences did not reach statistical significance.

When comparing the pediatric and geriatric groups, *E. coli* isolates from the pediatric group showed a higher susceptibility to nitrofurantoin (pediatric 99% vs. geriatric 97%; *p*>0.05) and fosfomycin (pediatric 99% vs. geriatric 92%; *p*<0.05). In contrast, sulfamethoxazole–trimethoprim susceptibility was slightly lower in pediatric *E. coli* isolates (pediatric 79% vs. geriatric 82%; *p*<0.05), but higher in pediatric *K. pneumoniae* isolates (pediatric 96% vs. geriatric 93%; *p*<0.05).

## 4. Discussion

### 4.1. Common microorganisms identified in urine culture

This study presented contemporary data on the distribution of microorganisms identified in urine cultures from pediatric and geriatric groups within community settings. Based on Table 1, the findings revealed that *S. saprophyticus* was commonly identified in the pediatric group but not in the geriatric group, indicating that it is a common uropathogen among young and sexually active females, as reported in previous studies.^10^,^11^ In contrast, *P. aeruginosa* was more prevalent in the geriatric group, consistent with its association with catheterization, which is more frequently performed in elderly patients in LTCFs than in their younger counterparts.^12^,^13^

Prescribers and antimicrobial stewardship teams should acknowledge the differences in microorganism distributions across age groups, as this may affect antimicrobial selection. This consideration is particularly important for pharmacists in BC, who are authorized to assess and prescribe for UTIs but are not yet permitted to order urine cultures and susceptibility testing in community settings.^14^,^15^ For instance, prescribers should be aware that elderly patients with UTIs unresponsive to nitrofurantoin, sulfamethoxazole–trimethoprim, and common oral β-lactam antimicrobials may have infections caused by *P. aeruginosa*, which has limited oral treatment options (Table 2). Notably, the presence of *S. agalactiae* in the genitourinary tract generally represents colonization rather than infection,^16^ but it has important implications for intrapartum management if identified during the prenatal period.^17^

### 4.2. Community pediatric and geriatric urinary antibiograms

Table 2 presents contemporary data on the antimicrobial susceptibility profiles of common uropathogens identified in urine specimens from pediatric and geriatric populations within community settings. These population-specific antibiograms support prescribing practices in pediatrics, where practitioners have expressed concern that relying on antimicrobial resistance patterns from adult patients or other countries may overestimate resistance.^7^ In addition, the antibiograms address the CLSI’s recommendation to develop antibiograms for patients aged 65 years and older in community settings, to meet prescribing needs in LTCFs.^1^ Moreover, these data benefit pharmacists who can prescribe empirical therapies for UTIs but are not currently authorized to order AST.^14^,^15^

By examining the distribution of common uropathogens and resistance trends in the community, prescribers can make informed decisions regarding empirical antimicrobial therapy—an area that remains insufficiently addressed in the guidelines issued by the government of BC.^3^ The guidelines recommend avoiding empirical fluoroquinolones, such as ciprofloxacin, due to moderate resistance rates and increased risk of adverse events, including tendinitis, central nervous system toxicity, aortic dissection, and *Clostridioides difficile* infection, all of which are of particular concern in elderly patients.^3^

In this study, the findings revealed that the effectiveness of ciprofloxacin declined with age: *E. faecalis* (pediatric 96.3% vs. geriatric 81.4%), *E. coli* (pediatric 73.7% vs. geriatric 67.3%), and *P. mirabilis* (pediatric 92.6% vs. geriatric 84.8%). Fluoroquinolone resistance may be partially attributable to prolonged antimicrobial exposure,^18^ which may explain why ciprofloxacin was less effective in the geriatric group, likely due to greater cumulative exposure over a lifetime compared to the pediatric group. This finding suggests that the empirical use of fluoroquinolones should be discouraged in elderly patients but not in the pediatric population, although fluoroquinolones have been associated with arthropathy in juvenile animal models.^19^,^20^ It is also important to note that fluoroquinolones are the only oral antimicrobial option for UTIs caused by *P*. *aeruginosa*, the sixth most common uropathogen identified in the geriatric group. Previous studies have suggested that UTIs caused by *P*. *aeruginosa* are often associated with elderly patients with indwelling urinary catheters,^12^,^13^ in which catheter removal or replacement may be indicated in addition to antimicrobial therapy.^21^

Interestingly, among the *E. coli* isolates, sulfamethoxazole–trimethoprim demonstrated higher susceptibility in the geriatric group. In contrast, among the *K. pneumoniae* isolates, sulfamethoxazole–trimethoprim exhibited higher susceptibility in the pediatric group. However, the reasons behind these opposing trends remain unclear. Therefore, further studies are warranted to determine whether the susceptibilities of sulfamethoxazole–trimethoprim differ consistently across age groups. Notably, fosfomycin exhibited >90% susceptibility in both pediatric and geriatric *E. coli* isolates. Nevertheless, studies are lacking to support the clinical efficacy of fosfomycin in children.^22^ Based on *in vitro* data alone, it is not possible to conclude whether fosfomycin would be a more effective antimicrobial in the pediatric group, despite its higher susceptibility rates compared with the geriatric antibiogram. Nitrofurantoin remained the most effective oral option for both pediatric and geriatric groups, with susceptibility rates of 99% and 97% (*p*<0.05), respectively. Although cefazolin also demonstrated >99% susceptibility across different microorganisms in both pediatric and geriatric groups, isolates were not tested when resistance patterns suggestive of ESBL were observed. This selective bias may underestimate the actual resistance rate in the community and is a well-recognized limitation of antibiograms.^1^

### 4.3. Comparison with other studies

In this study, the common uropathogens identified in both pediatric and geriatric groups align with previous reports, which consistently list *E. coli* as the most prevalent uropathogen.^23^ Nevertheless, there is a lack of studies that directly compare microorganisms and their susceptibilities between pediatric and geriatric populations. Some reports have suggested that the etiology of UTIs in elderly patients includes more gram-positive bacteria.^24^ However, that was not the case in the current study, as *S. saprophyticus*, a gram-positive bacterium, was more common in the pediatric group than in the geriatric group. A study of 212 elderly patients treated with ciprofloxacin demonstrated microbiological eradication and clinical resolution in 88.5% and 75.5% of patients, respectively, suggesting that ciprofloxacin was effective for the treatment of infections in elderly patients.^25^ The finding contradicts the results of the present study, which demonstrated lower ciprofloxacin susceptibility in the geriatric group compared with the pediatric group.

Nitrofurantoin remains an excellent oral antimicrobial option for cystitis associated with *E. coli* in hospital settings. Antibiograms from other hospitals in BC showed that nitrofurantoin and fosfomycin demonstrated >98%^26^-^28^ and >99%^29^ susceptibility, respectively, among *E. coli* isolates. In contrast, sulfamethoxazole–trimethoprim and ciprofloxacin demonstrated susceptibility rates of 75–84%^26^,^28^,^30^ and 64–77%,^26^,^28^,^31^ respectively. These susceptibility rates are consistent with the findings of the present study; however, the current study provides an additional breakdown of the susceptibility rates in pediatric and geriatric groups.

### 4.4. Strengths and limitations of the study

One major strength of the present study is the inclusion of data from a large community laboratory network, which provided contemporary evidence for prescribing in UTIs, a common condition in the community.^32^ The study included 373,068 urine specimens from patients of all ages and genders from 129 collection centers in BC, providing comprehensive and clinically relevant data. The study also compared the prevalence and susceptibility rates of microorganisms identified in urine specimens from both pediatric and geriatric groups within the same period, allowing a direct comparison of the prevalence and susceptibility patterns between the two groups.

A major limitation of the study is the exclusion of isolates with small sample sizes (*n* < 30) and antimicrobials not routinely tested, in accordance with the CLSI guidelines.^1^ These exclusions were intended to reduce selection bias that could mislead prescribing practices. The data from the present study should not be extrapolated to hospitals or communities in other geographic areas, and laboratories in these settings are encouraged to develop their own antibiograms and compare their findings with those of the present study. In addition, the microbiology laboratories did not have access to patients’ medical records and therefore could not determine whether the presence of microorganisms in urine indicated infection or colonization. Similar to prior studies,^2^ the present study did not investigate microorganisms present in insignificant numbers or mixed with at least two other types of microorganisms in urine culture, as these are deemed contaminants under standard laboratory practices.^33^ In addition, ESBL confirmatory testing was not routinely performed on isolates showing resistance patterns suggestive of ESBL, such as resistance to any of the third-generation cephalosporins. According to the 2025 CLSI M100 guideline on *Enterobacterales* AST, when using current breakpoints, routine ESBL testing is not necessary before reporting results.^9^ However, LifeLabs typically does not conduct routine cephalosporin AST when signs of ESBL are observed in susceptibility patterns. As shown in Table 2, not all isolates underwent cefazolin testing.

## 5. Conclusion

The common uropathogens identified in urine specimens and their susceptibilities differ between pediatric and geriatric groups, highlighting the importance of developing population-specific antibiograms in community settings. Ciprofloxacin demonstrated notably reduced susceptibility among uropathogens in elderly patients, whereas nitrofurantoin and fosfomycin demonstrated high susceptibility against *E. coli* in pediatric patients. In contrast, sulfamethoxazole–trimethoprim exhibited lower susceptibility to *E. coli* in children. Recognizing these age-related differences in antimicrobial resistance patterns is essential for guiding empirical therapy and optimizing antimicrobial stewardship efforts.

## Data Availability

Data used in this work are available from the corresponding author upon reasonable request.

## References

[1] Clinical &Laboratory Standards Institute. CLSI M39:Analysis and Presentation of Cumulative Antimicrobial Susceptibility Test Data. 5th ed;2022. Available from: https://clsi.org/standards/products/microbiology/documents/m39. [Last accessed on 2024 Jul 04]

[2] Mohammed RSD Yeung EYH Oral antimicrobial options for vancomycin-resistant *Enterococcus* isolates in urine culture. Bladder (San Franc) 2024;11 (2):e21200008. doi: 10.14440/bladder.2024.0016 39539468 10.14440/bladder.2024.0016 PMC11555133

[3] Government of British Columbia. Urinary Tract Infections in the Primary Care Setting –Investigation 2023. Available from: https://www2.gov.bc.ca/gov/content/health/practitioner-professional-resources/bc-guidelines/urinary-tract-infections. [Last accessed on 2025 Feb 23]

[4] Gajdács M Ábrók M Lázár A Burián K Urinary tract infections in elderly patients:A 10-year study on their epidemiology and antibiotic resistance based on the WHO access, watch, reserve (AWaRe) classification. Antibiotics (Basel) 2021;10 (9):1098. doi: 10.3390/antibiotics10091098 34572680 10.3390/antibiotics10091098 PMC8467796

[5] Pinnell R Ramsay T Wang H Joo P Urinary tract infection investigation and treatment in older adults presenting to the emergency department with confusion:A health record review of local practice patterns. Can Geriatr J 2021;24 (4):341–350. doi: 10.5770/cgj.24.518 34912489 10.5770/cgj.24.518 PMC8629500

[6] Ajayi T Radhakrishnan R Urinary tract infection in older adults in long-term care facilities. Can Med Assoc J 2016;188 (12):899–899. doi: 10.1503/cmaj.150708 26833739 10.1503/cmaj.150708 PMC5008938

[7] Le Saux N Antimicrobial stewardship in daily practice:Managing an important resource. Paediatr Child Health 2014;19 (4):261–265. doi: 10.1093/pch/19.5.261 24855430 10.1093/pch/19.5.261 PMC4029237

[8] Bailey P Antosz K Daniels R Gainey AB Burch AK Providing value to patients and providers via a pediatric statewide antibiogram in South Carolina. Antimicrob Stewardship Healthc Epidemiol 2023;3 (1):e78. doi: 10.1017/ash.2023.149 10.1017/ash.2023.149 PMC10127238 37113193

[9] Clinical &Laboratory Standards Institute. CLSI M100:Performance Standards for Antimicrobial Susceptibility Testing. 34th ed;2024. Available from: https://clsi.org/standards/products/microbiology/documents/m100. [Last accessed on 2024 Jul 04]

[10] Latham RH Running K Stamm WE Urinary tract infections in young adult women caused by *Staphylococcus saprophyticus*. JAMA 1983;250 (22):3063–3066 6644988

[11] Hovelius B Mårdh PA *Staphylococcus saprophyticus* as a common cause of urinary tract infections. Rev Infect Dis 1984;6 (3):328–337. doi: 10.1093/clinids/6.3.328 6377440 10.1093/clinids/6.3.328

[12] Mittal R Aggarwal S Sharma S Chhibber S Harjai K Urinary tract infections caused by *Pseudomonas aeruginosa*:A minireview. J Infect Public Health 2009;2 (3):101–111. doi: 10.1016/j.jiph.2009.08.003 20701869 10.1016/j.jiph.2009.08.003

[13] Inelmen EM Sergi G Enzi G When are indwelling urinary catheters appropriate in elderly patients?. Geriatrics 2007;62 (10):18–22 17922564

[14] Government of British Columbia. Pharmacy Services in B. C 2025. Available from: https://www2.gov.bc.ca/gov/content/health/accessing-health-care/pharmacy-services. [Last accessed on 2025 Feb 26]

[15] British Columbia Pharmacy Schedule. Endnote:B. C. Pharmacists Lab Services Referring Schedule 2024. Available from: https://www.bcpharmacy.ca/tablet/summer-24/endnote-lab-services-referring-schedule. [Last accessed on 2025 Jun 16]

[16] Martins ER Nascimento do ÓD Marques Costa AL Melo-Cristino J Ramirez M Characteristics of *Streptococcus agalactiae* colonizing nonpregnant adults support the opportunistic nature of invasive infections. Microbiol Spectr 2022;10 (3):e010822. doi: 10.1128/spectrum.01082-22 10.1128/spectrum.01082-22 PMC9241740 35604173

[17] Money D Allen VM The prevention of early-onset neonatal group B streptococcal disease. J Obstet Gynaecol Can 2016;38 (12):S326–S335. doi: 10.1016/j.jogc.2016.09.042 28063544 10.1016/j.jogc.2016.09.042

[18] Tam VH Louie A Fritsche TR Impact of drug-exposure intensity and duration of therapy on the emergence of *Staphylococcus aureus* resistance to a quinolone antimicrobial. J Infect Dis 2007;195 (12):1818–1827. doi: 10.1086/518003 17492598 10.1086/518003

[19] Burkhardt JE Walterspiel JN Schaad UB Quinolone arthropathy in animals versus children. Clin Infect Dis 1997;25 (5):1196–1204. doi: 10.1086/516119 9402381 10.1086/516119

[20] Adam D Use of quinolones in pediatric patients. Rev Infect Dis 1989;11 (Suppl 5):S1113–S1116. doi: 10.1093/clinids/11.supplement_5.s1113 2672245 10.1093/clinids/11.supplement_5.s1113

[21] Hooton TM Bradley SF Cardenas DD Diagnosis, prevention, and treatment of catheter-associated urinary tract infection in adults:2009 International Clinical Practice Guidelines from the Infectious Diseases Society of America. Clin Infect Dis 2010;50 (5):625–663. doi: 10.1086/650482 20175247 10.1086/650482

[22] Baquero-Artigao F Del Rosal Rabes T Fosfomycin in the pediatric setting:Evidence and potential indications. Rev Esp Quimioter 2019;32 (Suppl 1(Suppl 1)):55–61 PMC6555161 31131593

[23] Aspevall O Hallander H Gant V Kouri T European guidelines for urinalysis:A collaborative document produced by European clinical microbiologists and clinical chemists under ECLM in collaboration with ESCMID. Clin Microbiol Infect 2001;7 (4):173–178. doi: 10.1046/j.1198-743x.2001.00237.x 11422238 10.1046/j.1198-743x.2001.00237.x

[24] Shortliffe LMD McCue JD Urinary tract infection at the age extremes:Pediatrics and geriatrics. Am J Med 2002;113 (Suppl 1A):55S–66S. doi: 10.1016/s0002-9343(02)01060-4 12113872 10.1016/s0002-9343(02)01060-4

[25] Bianchi W Maggiolo F Ohnmeiss H Ciprofloxacin in elderly patients with underlying chronic diseases. J Int Med Res 1988;16 (5):386–393. doi: 10.1177/030006058801600508 3197916 10.1177/030006058801600508

[26] Vancouver Coastal Health. Vancouver General Hospital Antibiograms 2024 2025. Available from: https://aspires.vch.ca/antibiograms. [Last accessed on 2025 Jun 16]

[27] Fraser Health Authority. Nitrofurantoin:All Fraser Health (2023) 2025. Available from: https://app.firstline.org/en/clients/9-fraser-health/antimicrobials/1895-nitrofurantoin/antibiograms/1792-all-fraser-health-sites?navigator=eJylj0EKg0AMRa9SsmrBUhQK7ey8QC8gIqOd2oAmkplZiXfX6UqtFKSrkPzPy_9ZD7YSY6gg3RpQ8OZpRFAL-86CyvIhWls0OWyxEi5RN3YyO3RNENK1gBUTqB7wCSpOkmEHd4Z9oBN-eZl0RtrEdlp0a8NtASmCA-Lb_XqmJWRPlEvYSuT68-NHLjHOCxXb-uGYzjCnrxrx_zXyEee_oek%3D. [Last accessed on 2025 Jun 16]

[28] Interior Health. Interior Health Antibiogram 2024 2025. Available from: https://www.interiorhealth.ca/sites/default/files/PDFS/antimicrobial-susceptibility-report.pdf. [Last accessed on 2025 Jun 16]

[29] Fraser Health Authority. Fosfomycin:All Fraser Health (2023) 2025. Available from: https://app.firstline.org/en/clients/9-fraser-health/antimicrobials/1925-fosfomycin/antibiograms/1792-all-fraser-health-sites?navigator=eJyljrEKwjAQhl9FblKoSAIOZuviS5QS0pjWgyZXknSQ0ne3cbG2OhSn4-7_7-MrBgjaG-OkU9aAgDtNI4PGU98FEEU5ZsuKchEtak8VqjZM5YixTUG-DFCTAzEA3kAwzscN3Bn2SqEm-9DoviI75ZUN6fYBkKkB7MLPx_oN2KJwSluF1Lz4P3y8ib13cp3t9vns_bBSZ_-pl0921JkE. [Last accessed on 2025 Jun 16]

[30] Fraser Health Authority. Cotrimoxazole:All Fraser Health (2023) 2025. Available from: https://app.firstline.org/en/clients/9-fraser-health/antimicrobials/2011-cotrimoxazole/antibiograms/1792-all-fraser-healt h-sites?navigator=eJylj8EKgzAQRH-l7KkFSxuPuUk_QyTENNgFk5VNhFLx32t6UmsP0tOyO8Pb mXKAYNhar7x2F iQ8aBoZNE x9F0CW1ZitLdp HdGiYatRt mMwRY5uEYi2gIQ9yALyDFHk-7uDOsDeKjI6e-kWt3aR2mrUL6bZgqOSA_CrE2SwYe4Jc0lYjNZ8Xv1OxjT17tSkfjsUMcvrqIP7uUL0BZn-fiQ%3D%3D. [Last accessed on 2025 Jun 16]

[31] Fraser Health Authority. Ciprofloxacin:All Fraser Health (2023) 2025. Available from: l7KkFSzH00tyknyEiMU3tQszKJoIg_ntNT2rtQXpadmd4O5MP4DUb40qnGgMS XjSNBGqmrvUg82JM1hblAjaomSpU1k_mgMFGIVsLqMmBHAAfIFMhxh3cGfaOLdPTUq80uk1qq 1g1PtWjDI6IL1dxVkvGHuCXOJWIdWfF79TsQkdu 3JTPhyzGeT01SH9u0PxBh VdnwY. [Last accessed on 2025 Jun 16]

[32] Tüzün T Sayın Kutlu S Kutlu M Kaleli İ Risk factors for community-onset urinary tract infections caused by extended-spectrum b-lactamase-producing Escherichia coli. Turk J Med Sci 2019;49 (4):1206–1211. doi: 10.3906/sag-1902-24 31385490 10.3906/sag-1902-24 PMC7018203

[33] Miller JM Binnicker MJ Campbell S Guide to utilization of the microbiology laboratory for diagnosis of infectious diseases:2024 update by the infectious diseases society of America (IDSA) and the American Society for Microbiology (ASM). Clin Infect Dis 2024 ciae104. doi: 10.1093/cid/ciae104 38442248 10.1093/cid/ciae104

